# Cesarean section and the risk of allergic rhinitis in children: a systematic review and meta-analysis

**DOI:** 10.1038/s41598-023-44932-8

**Published:** 2023-10-26

**Authors:** Zixin Liu, Li Xie, Xiaohua Liu, JunRong Chen, Yaqian Zhou, Jialin Zhang, Honghui Su, Yide Yang, Mei Tian, Jian Li, Yunpeng Dong

**Affiliations:** 1 Key Laboratory of Model Animals and Stem Cell Biology in Hunan Province, School of Medicine, Hunan Normal University, 410013 Hunan, China; 2 Third Xiangya Hospital, Central South University, 138 Tongzipo Road, 410013 Changsha, Hunan China; 3 Key Laboratory of Study and Discovery of Small Targeted Molecules of Hunan Province, School of Medicine, Hunan Normal University, 410013 Changsha, Hunan China; 4 Second Xiangya Hospital, Central South University, 139 Renmin Road, 410011 Changsha, Hunan China; 5 The Engineering Research Center of Reproduction and Translational Medicine of Hunan Province, Hunan Normal University, 410013 Changsha, Hunan China; 6 Department of Otolatyngoloty-Head and Neck Surgery, Yichang Central People’s Hospital, Three Gorges University, 443000 Hubei, China

**Keywords:** Paediatrics, Public health

## Abstract

Multiple evidence indicates that perinatal factors make impact on immune development and affect offspring allergic rhinitis (AR) risk. In this systematic review and meta-analysis, we examined available published studies to clarify the relationship between cesarean section (C-section) and offspring AR in children. To explore the relationship between C-section, especially the special attention was paid to different cesarean delivery mode, and the risk of AR in children. Articles were searched using PubMed, Web of Science, EMBASE, Cochrane Library, China knowledge Network, Wanfang, and China Science and Technology Journal databases. A meta-analysis of 22 studies published before August 1, 2022, which included 1,464,868 participants, was conducted for statistical analysis with RevMan5.4. The correlation strength between C-section and offspring AR was determined by combining odds ratio (*OR*) and 95% confidence interval (*95% CI*). Meta-regression and subgroup analyses were used to explore potential sources of heterogeneity. Publication bias was detected using the funnel chart and Egger tests. Meta-analysis revealed that there was a significant correlation between C-section and children AR (*OR* = 1.19, *95% CI*: 1.12–1.27, *P* < 0.001), especially C-section with a family history of allergy (*OR* = 1.82, *95% CI*: 1.36–2.43, *P* < 0.001). Moreover, elective C-section (without genital tract microbe exposure) had the higher risk of offspring AR (*OR* = 1.24, *95% CI*: 1.05–1.46, *P* = 0.010) compared with the whole study. Meta-regression demonstrated that sample size explained 38.0% of the variability between studies, and year of publication explained 18.8%. Delivery by C-section, particularly elective C-section and C-section with a family history of allergy can increase the risk of AR in children.

## Introduction

Allergic rhinitis (AR)^1^ is an IgE-mediated inflammation of the nasal mucosa, with symptoms including excess tear production, runny nose, and sneezing. Studies have reported that the global prevalence of AR is rising^2^ with a prevalence of 10%–30% in the United States. However, relatively few studies reported the incidence of AR in children. The International Childhood Asthma and Allergy in Childhood (ISAAC)^3^ estimated the prevalence of allergic diseases in different age groups through a multi-centered global survey, and reported prevalence rates of 8.3% and 15.1% for those aged 6–7 and 13–14 years, respectively. A meta-analysis of all the recent studies conducted under the ISAAC protocol (1,430,329 children aged 0–18 years) reported^4^ an overall AR prevalence of 12.66%. Currently, many regional studies reported the prevalence of childhood AR in China, varying from 12.08 to 30.04% in every city^5^. It is important to note that AR is more than just sneezing and a nuisance for the children. AR may contribute to sleep loss, learning impairment, decreased overall cognitive functioning, aggravation of underlying asthma, and increased propensity to develop asthma^6,7^.

According to the recommendation of the World Health Organization (WHO), the cesarean section (C-section) rate should be controlled at 10–15%^8^. However, women's preference for C-section has increased due to concerns of the short- and long-term risks related to vaginal delivery (VD)^9^. Presently, the C-section rate worldwide continuously exceeds the WHO recommendation^10^. The national epidemiological survey of the C-section rate in China reported an annual increase in the domestic C-section rate, reaching 34.9%^11^. C-section can be a life-saving intervention when medically indicated, but this procedure can also lead to short-term and long-term health effects for women and children. C-section is not only associated with an increased risk of uterine rupture, abnormal placentation, ectopic pregnancy, stillbirth, and preterm birth in woman, but also associated with altered immune development in children, like allergy, atopy, and asthma, and reduced intestinal gut microbiome diversity in children^12^.

Correlation between C-section and AR risk in future generations have attracted much attention. A previous meta-analysis of C-section and offspring AR was conducted in 2009^13^, which included only seven studies and revealed a positive correlation between C-section and offspring AR. After that, more and more studies still further focus on this topic and have assessed the association between mode of delivery and AR in the following years. Many studies have explored the relationship between C-section and the incidence of AR in children. Because a discrepancy in quantity and degree, the results are not consistent. Some previous studies showed that C-section is not associated with the risk of AR in children^14–16^, Others showed that C-section^17–20^, especially C-section with a family history of allergy^21–23^ could increase the risk of AR in children. More recently, studies linking the risk of AR to mode of delivery have examined the roles of emergency C-section after the onset of labour, versus elective prelabour C-section. Therefore, whether C-section increases the risk of offspring AR remains controversial^24^. Moreover, the previous meta-analysis^13^ included people of all ages including adults and children. However, the risk of AR in adults is often related to smoking, long-term exposure to adverse environments, occupational factors and so on^25,26^. Our study concentrated on the effects of birth-mode changes on AR in children, and more on early-life observed effects. In line with the ‘hygiene hypothesis’, the intestinal microflora may be modified in those children who are not exposed to the vaginal microbiota, but the effect decreases with the age of the offspring^27^. The mode of delivery is an important factor affecting the composition of intestinal flora in newborns and infants. Caesarean section may negatively affect the normal development of the microbiota, leading to decreased amounts of lactobacilli and bifidobacteria and increased amounts of Clostridia^28^. Reduced diversity of the intestinal microbiota during infancy is associated with increased risk of allergic disease at children^29^. To address this problem, we conducted a systematic review and meta-analysis of epidemiological studies to explore the relationship between C-section, especially the special attention was paid to different cesarean delivery method, and the risk of AR in children.

## Materials and methods

### Protocol and registration

This systematic review was registered on PROSPERO (CRD: 42021251713) and conformed to the MOOSE^30^ and Preferred Reporting Items for Systematic Reviews and Preferred Reporting Items for Systematic Reviews and Meta-Analyses (PRISMA) guidelines^31^.

### Information sources and search strategy

We searched international online databases, such as PubMed, EMBASE, Web of Science, CNKI, and VIP and Wanfang. In PubMed, the searches were carried out with a combination of subject and free words. The search strategy was designed and set up by an expert in database searches (Yide Yang) and two investigators (Zixin Liu and Junrong Chen).We have arranged the search terms according to PICO model to provide more details to get more accurate search results. According to the search subject of this article. Search terms used were as follows: Patient: pregnant women, pregnant, minors, adolescent, offspring , early life , toddler, child; Intervention: cesarean section, c-section, abdominal deliveries, post-cesarean section; Comparison: natural childbirth, vaginal delivery; Outcome: allergic rhinitis, hay fever, allergic rhinoconjunctivitis, seasonal allergic rhinitis. The titles and abstracts of the studies were screened, and full papers were retrieved if a decision could not be made based on the abstract alone. To identify all potentially eligible studies, the reference lists of all comprised studies were examined. We searched from the beginning of databases up to August 1, 2022.

### Inclusion and exclusion criteria

The inclusion criteria were as follows: Study population: children aged 0–18 years old. Study types: cohort studies, cross-sectional studies, and case–control studies. Exposure factors: children delivery through C-section; Outcome: offspring diagnosed with AR; and relative risk (*RR*), hazard ratio (*HR*),or odds ratio (*OR*) and their confidence intervals can be obtained, or enough data to calculate them.

The exclusion criteria were as follows: literature with incomplete relevant data and when the original data could not be obtained from the author; non-Chinese and non-English literature; case studies, systematic reviews, dissertations, meta-analyses; and low-quality research.

### Data extraction

All studies were included in this systematic review if they fulfilled the inclusion criteria. After an initial sweep, the two investigators (Zixin Liu and Junrong Chen) independently performed a thorough title and abstract screening. Articles were added to this shortlist of studies through reference cross-checking. Disagreements were resolved by a consensus discussion in a meeting with the senior author (Jian Li). After agreement between the two investigators, studies were merged into a final list for full-text analysis and data extraction. Both investigators agreed on the final shortlist for data analysis and extraction.

### Data collection process

Two researchers (Zixin Liu, Junrong Chen) independently examined the literature and extracted the data. In the case of disagreement, a third researcher (Jian Li) was consulted if necessary. The final determination of the extracted literature was the result of a consensus reached by three individuals. If there were significant doubts regarding the data from a particular study, the corresponding authors were contacted for confirmation of the extracted data. Information extracted from the tables in the literature was as follows: study details (author, year of publication, and journal name); study methodology and characteristics; study design (case–control study, cross-sectional study, and cohort study); participant information (age, sex, country or territory, sample size, diagnosis, parental atopy history, and type of C-section); follow-up or study duration, relevant independent, and dependent variables; data analysis methods; adjustment for confounding factors; and effects obtained from study results (studies offered available data on the relevant risk estimates including an odds ratio [*OR*] and their 95% confidence interval [95% *CI*], or enough data to compute them). If studies reported relative risks (*RR*) or hazard ratios (*HR*) as the effect size, Hazard ratios were directly considered as *RR*^32^. *RR* (*95% CI*) were converted to *OR* (*95% CI*) by using the following formula: *OR* = [(1-P_0_) * *RR*/ (1-*RR* * P_0_)], where P_0_ indicates the incidence of the outcome of interest in the non-exposed/reference group. The standard error of the resulting converted relative risk was then determined with this formula: SElog (*RR*) = SElog (*OR*)*log (*RR*)/log (*OR*)^33^. This meta-analysis used OR as the effect size because some research reports only provided OR and did not include HR or RR. In such situations, using OR as the effect size allowed for better utilization of the data for comprehensive analysis. Adopting a unified algorithm and interpretation method could simplify the process of data integration and comparison, and improve the reliability and interpretability of the results. When several estimates were reported within the same study, the most adjusted model was used for the pooled analysis.

### Quality assessment

For cohort and case–control studies, the Newcastle–Ottawa Scale (NOS)^34^ was used to assess the study quality, and the total NOS score was 10. NOS was used to evaluate case–control (selection, comparability, and exposure) and cohort studies (selection, comparability, and results). Those with scores of ≤ 5 and ≥ 6 were classified as low- and high-quality studies, respectively. For cross-sectional studies, 11 checklists recommended by the Agency for Healthcare Research and Quality^35^ were used to evaluate the studies^36,37^. A higher total score of each item corresponded to a higher-quality document; if the answer was "no" or "unclear,” the item score was "0," and if the answer was "yes," the item score was "1," of which the fifth was a reverse score. The evaluation of literature quality was as follows: low quality = 0–3, medium quality = 4–7, and high quality = 8–11^38^. The studies were independently evaluated by two researchers (Zixin Liu and Junrong Chen). In case of differences, a third experienced investigator (Jian Li) was consulted until a consensus was reached.

### Data analysis

RevMan5.4 and STATA16.0 was used for statistical analysis, and *OR* was used as the effect index. The correlation strength between C-section and offspring AR was determined by combining *OR* and 95% *CI*. Cochran’s Q test and I^2^ index were used to evaluate the heterogeneity of the studies^39^. Regarding the heterogeneity test, a *P*-value of ≥ 0.1 and I^2^ of ≤ 50% suggested homogeneity among the studies, and a fixed effects model was used for merging; a *P*-value of ≤ 0.1 and I^2^ of ≥ 50% suggested heterogeneity among the studies, and a random effects model was used to estimate the combined effect. Prediction intervals were calculated in meta-analyses^40^ with at least four studies^41^ to take the large between-study heterogeneity into account. Meta-regression was used to investigate potential sources of heterogeneity between the studies. For analytical purposes, the studies’ characteristics were grouped as follows: study design; study areas; year of publication; sample size; age; diagnostic mode and follow-up period. According to the included studies, a subgroup analysis was also performed on type of C-section and C-section with or without history of atopy. Publication bias was assessed using funnel plot and Egger’s test correlation. Sensitivity analysis was performed by excluding the study one by one. *P* < 0.05 was deemed statistically significant.

## Results

### Study retrieval steps and screening results

**Figure 1:**
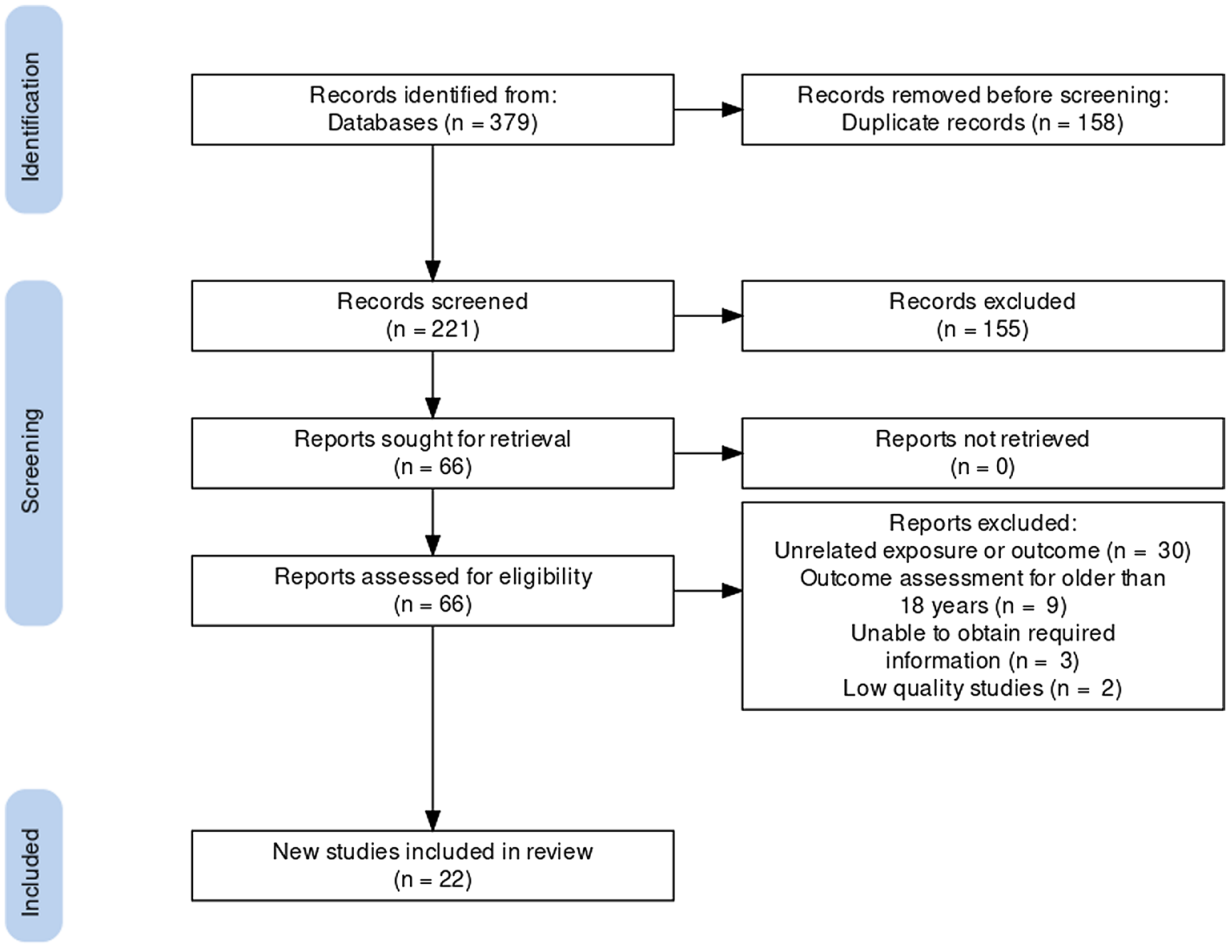
Literature screening process and results.

Two reviewers independently screened the studies included in the meta-analysis. We calculated the percentage agreement to evaluate the consistency of the two reviewers in the search process for the meta-analysis. The first round of percentage agreement was 86.7%. After discussing with a third experienced expert, the second round of percentage agreement improved to 100%. We retrieved 379 citations from multiple electronic databases. After excluding 158 repetitive studies, and reading titles and abstracts, 155 studies were excluded because they were systematic reviews, reviews, letters, case reports, and study factors were not relevant. After evaluating 66 full-text articles, studies with an outcome index of > 18 years (n = 9), lack of focus on the between C-section and childhood AR (n = 30), those that could not obtain specific data (n = 3), and low quality studies (n = 2) were excluded. Finally, 22 articles were included in this study^14–23,42–53^. The screening process and results are shown in Fig. 1.

### Basic characteristics and quality evaluation of studies

This study included 22 studies involving 1,464,868 participants. Regarding research methods, there were 11 cohort studies^14,16,18–20,23,42–44,48,51^, 2 case–control studies^21,49^, and 9 cross-sectional studies^15,17,22,45–47,50,52,53^. For study areas, 6 were conducted in Europe^14,17,18,42–44^, 9 in Asia^16,21,45–50,52^, 5 in North America^19,20,23,51,53^, and 2 in South America^15,22^. In previous studies, Richards^51^ used *RR* as the effect value, Mitselou^18^ used *HR*. We ensured the comprehensiveness of analysis and the maximization of statistical ability by calculating *OR*^54^ as the combination effect value^55^.

The diagnosis of AR included the questionnaire survey and clinical diagnosis. In the questionnaire survey, 5^15,22,46,52,53^ studies were conducted based on the ISAAC questionnaire. Clinical diagnosis was based on the physician's judgment based on the clinical history, using International Classification of Disease (ICD) diagnosis codes or Positive Skin Prick Test (SPT) reaction from the electronic health records.

Cohort studies were mainly from Europe and North America, while cross-sectional and case–control studies were concentrated in Asia. Pistiner 's study^23^ examined the relation between mode of delivery and the development of AR with a parental history of atopy, 3^21,22,45^ studies used stratified analysis of C-section according to family history of allergies,3^18,19,51^ studies reported the effect of different types of C-section on AR in children. The most adjusted covariates included age, preterm birth or neonatal complications, educational level, income, and birth order. The general characteristics and quality evaluation results were shown in Table 1.

**Table 1:** Characteristics of studies and quality evaluation. ISAAC = international study of asthma and allergies in childhood, BMI = body mass index, *OR* = odds ratio.

Author	Country	Study design	Sample size	Age (y)	Diagnostic mode	Adjusted for confounders
Nafstad 2000^42^	Norway	Cohort Study	3,754	0–4	Clinical diagnosis	Crude *OR*
Mckeever 2002^43^	England	Cohort Study	24,690	0–11	Clinical diagnosis	Sex, age(y), prematurity, parental atopy history, parental smoking behavior, maternal age, general practice
Negele 2004^44^	Germany	Cohort Study	2,500	0–2(< 2)	Questionnaire (self-made), Clinical diagnosis	sex, study area, parental atopy history, maternal smoking, breast-feeding
Renz-Polster 2005^19^	America	Cohort Study	8,953	3–10	Clinical diagnosis	Sex, age at diagnosis, birthweight, birth order, exposure to antibiotics in the postpartum period, ethnicity, multiple gestation, maternal age, education, marital status, smoking status during pregnancy, use of asthma and/or hay fever medications
Salam 2006^20^	America	Cohort Study	3,464	8–17	Questionnaire (self-made)	Age, sex, race, birth order, birth weight, community of residence, parental education, health insurance coverage, parental atopy history, exposure to in utero smoking, maternal age at childbirth, requirement for special care after birth, calendar period of birth, and history of pneumonia, bronchitis, bronchiolitis, and croup
Pistiner 2008^23^	America	Cohort Study	432	0–9(< 9)	Questionnaire (self-made), Clinical diagnosis	Age and sex, birth weight, Endotoxin levels, ≤ 3 Episodes of nasal catarrh in the first year of life, day care attendance in the first year of life
Park 2010^45^	South Korea	Cross-Sectional Study	279	< 6	Clinical diagnosis	Sex, age, gestational age, birth weight, breast-feeding
Huang 2014 ^21^	China	Case–control Study	854	5–14	Clinical diagnosis	Sex, gestational age, birth weight, breast-feeding, family smoking before and after birth, family smoking at present, housing floors, domestic pets, mildew and carpet in the home, antibiotic use within 1 year old, parental atopy history
Li 2015^46^	China	Cross-Sectional Study	20,803	5–13	Questionnaire(ISAAC)	Age, sex, family income, family numbers, resident area per capita, parental education level, city
Brandao 2016^22^	Brazil	Cross-Sectional Study	672	0–6(< 6)	Questionnaire(ISAAC)	Gestational age, birth weight, breast-feeding, mother’s smoking during pregnancy, family income, mother’s schooling level, parity, number of persons who sleep in the room with the child, attendance to nursery to the age of 2 years, pneumonia ever
Chu 2017^47^	China	Cross-Sectional Study	12,639	5–12	Questionnaire (self-made)	Maternal education level, paternal education level, maternal history of diabetes in pregnancy
Loo 2017 ^48^	Singapore	Cohort Study	1,237	0–5(< 5)	Questionnaire (self-made), Clinical diagnosis	Age, ethnicity, educational level, parity, parental atopy history, gestational diabetes mellitus status, early pregnancy body, offspring sex, gestational age
Gerlich 2018^14^	Germany	Cohort Study	1,006	9–11	Questionnaire(ISAAC) , Clinical diagnosis	Sex, parental education, study location, place of birth, parental atopy history, presence of siblings, maternal smoking during pregnancy, season of birth
Krzych-Fałta 2018^17^	Poland	Cross-Sectional Study	9,231	6–7 13–14	Questionnaire(ISAAC) , Clinical diagnosis	Unknown
Zeng 2018^49^	China	Case–control Study	296	4–14	Clinical diagnosis	Parental atopy history, domestic animals, disease duration ≥ 24 months, asthma, seafood consumption, second-hand smoke smoking, breast-feeding
Han 2019^16^	South Korea	Cohort Study	1,374	4–12	Clinical diagnosis	Age and sex, breast-feeding, number of siblings, parental atopy history, living area
Yu 2019^50^	China	Cross-Sectional Study	183,449	6–18	Questionnaire (self-made)	Age, Year, sex, breast-feeding, father smoking
Gorris 2020^15^	Ecuador	Cross-Sectional Study	400	3–12	Questionnaire(ISAAC)	Crude* OR*
Mitselou 2020^18^	Sweden	Cohort Study	1,059,600	0–13	Clinical diagnosis	Sex, maternal age at delivery, country of birth, parity, maternal BMI, early pregnancy smoking and parental atopy history
Richards 2020^51^	America	Cohort Study	117,768	6–10	Clinical diagnosis	Maternal age, education, race, pre-pregnancy BMI, smoking, antibiotics during pregnancy, parental atopy history, sex, gestational age, birth weight, NICU admission, birth order, breast-feeding
Hu 2021^52^	China	Cross-Sectional Study	10,464	6–11	Questionnaire(ISAAC)	Sex, age, socioeconomic status, only one child in the household, parental atopy history
Meza-Lopez 2021^53^	Mexico	Cross-Sectional Study	1,003	6–7, 13–14	Questionnaire(ISAAC)	Unknown

### Relationship between C-section and offspring AR

**Figure 2:**
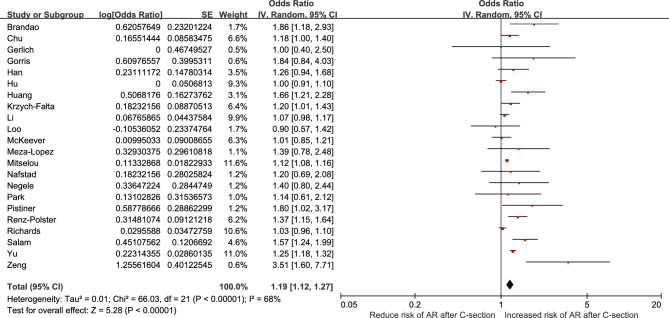
Meta-analysis of the effect of cesarean section and children allergic rhinitis, random effect model.

In this meta-analysis, the rate of heterogeneity was high (I^2^ = 68%, *P* < 0.001). A random effects model was used to demonstrate the results of the meta-analysis, and revealed that there was a significant correlation between C-section and children AR (*OR* = 1.19, *95% CI*: 1.12–1.27, *P* < 0.001). The results were shown in Fig. 2.

### Subgroup analysis and meta-regression

A series of random-effects subgroup analyses and meta-regression was conducted to examine whether the association between C-section and AR. For our study, the associations between C-section and AR were not substantially altered by study design (cohort study/cross-sectional study/ case–control study), study areas (Europe/Asia/North America/South America), age (< 6, ≥ 6, both), diagnostic mode (self-made questionnaire, ISAAC questionnaire, clinical diagnosis, questionnaire & diagnosis), and follow-up period (N/A, < 5, ≥ 5). However, meta-regressions confirmed the year of publication (2000–2009, 2010–2019, 2020–2022), and sample size (< 1000, 1000–9999,  ≥ 10,000) may have been the possible causes of heterogeneity.

In the meta-regression analysis, sample size (38.0%) and year of publication (18.8%) explained the variability between studies. Subgroup analysis showed that, a greater effect size was observed for articles published before 2020 (1.32[1.09–1.60] and 1.24 [1.12–1.37]) compared with the whole study [1.19(1.12–1.27)], and the difference was statistically significant in the meta-regression (*P* = 0.031). A larger effect size of studies with sample sizes less than 10,000 (1.75 [1.42–2.15] and 1.30 [1.18–1.43]) was found in comparison with studies model assessment of sample size greater than 10,000 (1.10 [1.03–1.17]), and the difference was also significant (*P* = 0.001). Subgroup analysis indicated that for most of the variables evaluated, the association measurement continued to be associated with AR. Subgroup analysis and meta-regression results were shown in Table 2.

**Table 2:** Subgroup analysis and meta-regression of the effect measurement. ISAAC = international study of asthma and allergies in childhood, *OR* = Odds Ratio.

Variable	N	Sample size	*OR* (*95% CI*)	*P*	Heterogeneity (confidence limits)	Meta-regression *P* value
All studys	22	1,464,868	1.19(1.12–1.27)	< 0.01	68.2% (55.6–79.5%)	N/A
Study design						
Cohort study	11	1,224,778	1.17(1.07–1.27)	< 0.01	59.4% (21.2–79.1%)	0.445
Cross-Sectional study	9	238,940	1.17(1.06–1.29)	< 0.01	67.6% (34.7–83.9%)	
Case–control study	2	1,150	2.21(1.08–4.50)	0.03	66.6% (N/A)	
Test for subgroup differences: Chi^2^ = 3.04, df = 2 (*P* = 0.22), I^2^ = 34.3%						
Study areas						
Europe	6	1,100,781	1.12(1.08–1.16)	< 0.01	0% (0–74.62%)	0.313
Asia	9	213,195	1.18(1.06–1.33)	< 0.01	75.5% (52.8–87.3%)	
North America	5	131,620	1.34(1.06–1.70)	0.01	81.4% (57.0–92.0%)	
South America	2	1,072	1.85(1.25–2.75)	< 0.01	0% (N/A)	
Test for subgroup differences: Chi^2^ = 9.01, df = 3 (*P* = 0.03), I^2^ = 66.7%						
Year of publication (y)						
2000–2009	6	43,793	1.32(1.09–1.60)	< 0.01	57.8% (0–82.9%)	**0.031**
2010–2019	11	231,840	1.24(1.12–1.37)	< 0.01	60.3% (23.1–79.5%)	
2020–2022	5	1,189,235	1.07(0.99–1.15)	0.08	60.6% (0–85.2%)	
Test for subgroup differences: Chi^2^ = 7.76, df = 2 (*P* = 0.02), I^2^ = 74.2%						
Sample size						
< 1000	6	2,933	1.75(1.42–2.15)	< 0.01	1.1% (0–13.1%)	**0.001**
1000–9999	9	32,522	1.30(1.18–1.43)	< 0.01	0% (0–64.8%)	
≥ 10,000	7	1,429,413	1.10(1.03–1.17)	< 0.01	78.8% (56.3–89.7%)	
Test for subgroup differences: Chi^2^ = 22.71, df = 2 (*P* < 0.0001), I^2^ = 91.2%						
Age (y)						
< 6	5	8,442	1.27(0.98–1.65)	0.07	22.3% (0–67.7%)	0.869
≥ 6	7	326,385	1.17(1.04–1.32)	0.01	82.1% (64.2–91.0%)	
Both	10	1,130,041	1.21(1.10–1.33)	< 0.01	65.7% (32.8–82.5%)	
Test for subgroup differences: Chi^2^ = 0.42, df = 2 (*P* = 0.81), I^2^ = 0%						
Diagnostic mode						
Questionnaire (self-made)	3	199,552	1.28(1.14–1.44)	< 0.01	49.7% (N/A)	0.360
Questionnaire (ISAAC)	5	33,342	1.13(0.97–1.32)	0.11	59.9% (0–85.0%)	
Clinical diagnosis	9	1,217,568	1.18(1.07–1.30)	< 0.01	69.8% (39.8–84.8%)	
Questionnaire and diagnosis	5	14,406	1.20(1.04–1.40)	0.01	0% (0–79.42%)	
Test for subgroup differences: Chi^2^ = 1.90, df = 3 (*P* = 0.59), I^2^ = 0%						
Follow-up period (y)						
N/A	11	240,090	1.23(1.11–1.38)	< 0.01	72.8% (50.2–85.2%)	0.355
< 5	6	44,735	1.28(1.09–1.50)	< 0.01	51.7% (0–80.6%)	
≥ 5	5	1,180,043	1.18(1.00–1.18)	0.06	51.5% (0–82.2%)	
Test for subgroup differences: Chi^2^ = 5.13, df = 2 (*P* = 0.08), I^2^ = 61.0%						

Significant values are in bold.

### Results of the subgroup analysis according to the family history of allergy

**Figure 3:**
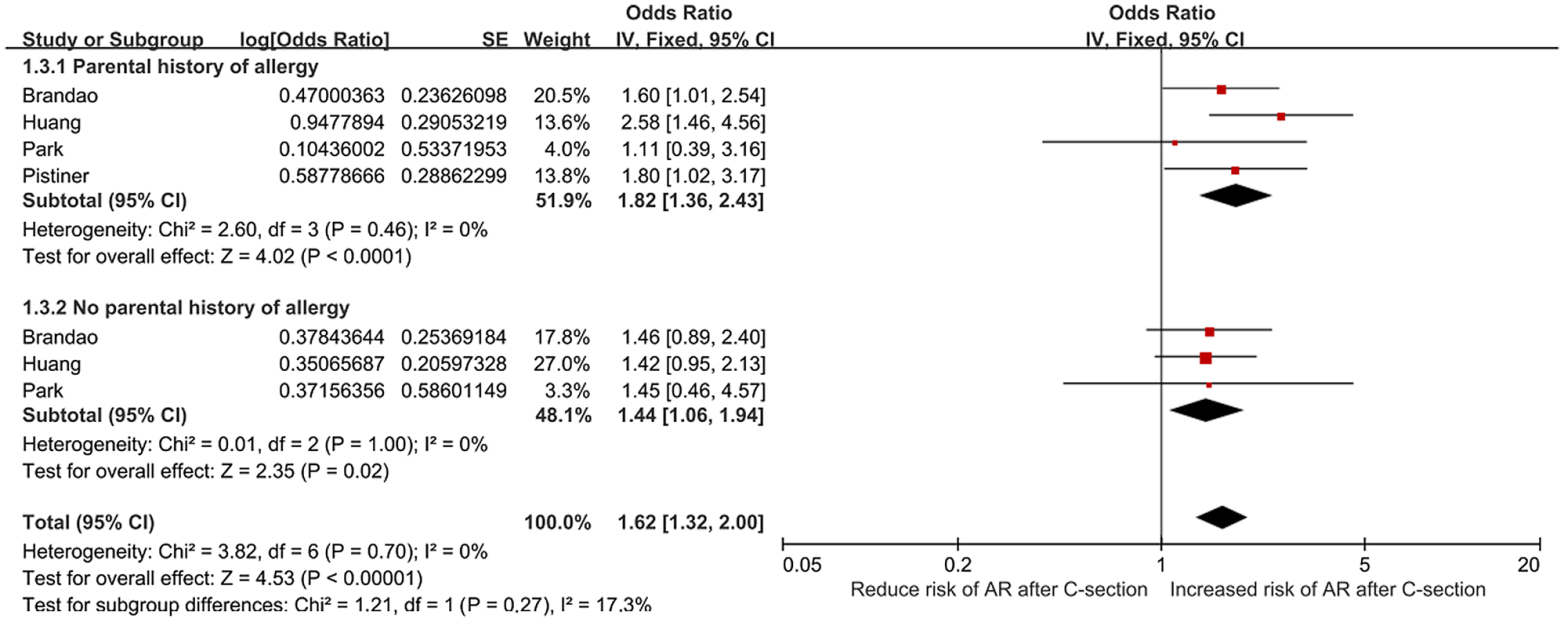
Relative risk between cesarean section with family history of allergy and allergic rhinitis in children, fixed effects model.

Four studies were incorporated. The result demonstrated that the heterogeneity was low (I^2^ = 0%, *P* = 0.700). A fixed effects model was used to estimate the combined effect. The meta-analysis demonstrated that C-section with parental history of allergy had a higher risk of offspring AR (*OR* = 1.82, *95% CI*: 1.36–2.43, *P* < 0.001) compared with C-section with No parental history of allergy (*OR* = 1.44, *95% CI*: 1.06–1.94, *P* = 0.020) and the whole analysis (*OR* = 1.19, *95% CI*: 1.12–1.27, *P* < 0.001) as shown in Fig. 3.

### Results of the subgroup analysis according to C-section type

**Figure 4:**
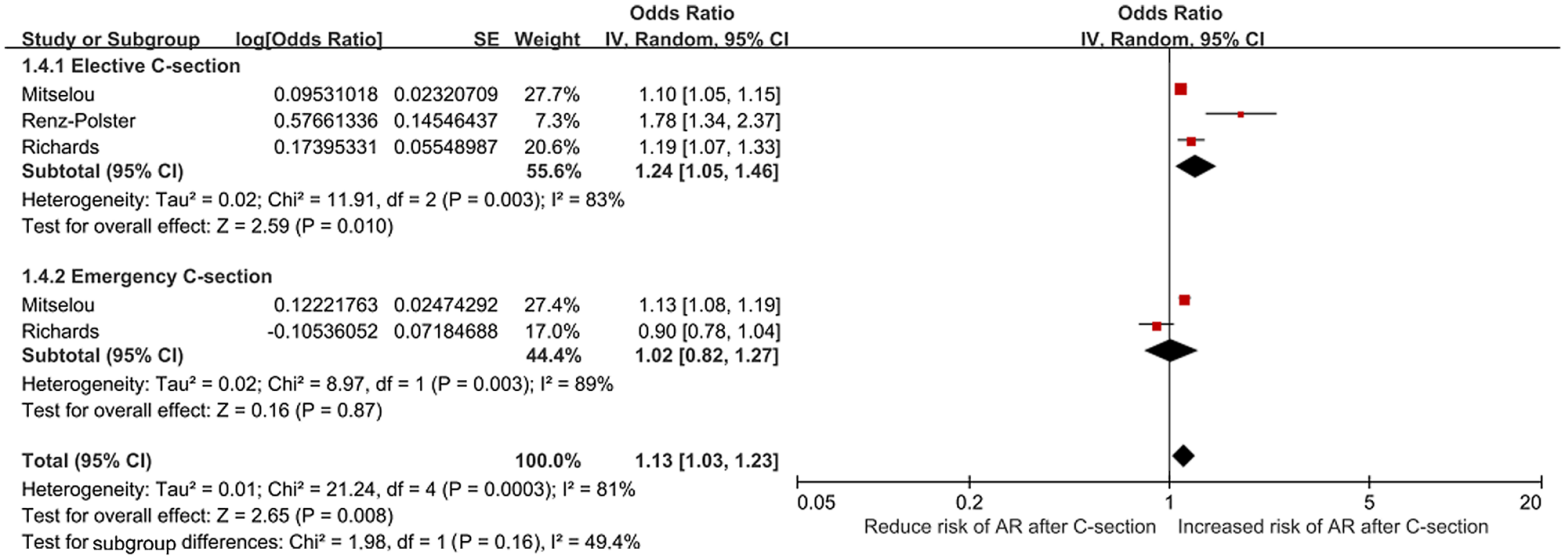
Relative risk between cesarean section Type and allergic rhinitis in children, random effects model.

Emergent C-section, means a period of vaginal delivery and with genital tract microbe exposure but terminated because iatrogenic reason, was showed significantly higher risk for children AR than elective C-section in Mitselou’s^18^ study. But Richards’s^51^ study showed not significant (*OR* = 0.90, *95% CI*: 0.78–1.04). So meta-analysis was conducted and showed that elective C-section (without genital tract microbe exposure) had the higher risk of offspring AR (*OR* = 1.24, *95% CI*: 1.05–1.46, *P* = 0.010) compared with the whole analysis (*OR* = 1.19, *95% CI*: 1.12–1.27,* P* < 0.001). In our research, no association between emergency cesarean (with genital tract microbe exposure) delivery and AR in children was found (*OR* = 1.02, *95% CI*: 0.82–1.27,* P* = 0.870). The results were shown in Fig. 4.

### Publication bias analysis and sensitivity test

The funnel chart was drawn using Stata16.0, and the trim-and-fill method was used to calculate the combined estimate after adjusting for publication bias. The resulting estimate did not change significantly. The statistical tests of publication bias were not significant for the *OR* of AR in the overall C-section (Egger’s test: *p* = 0.137), cohort study (Egger’s test:* p* = 0.390), cross-sectional study (Egger’s test: *p* = 0.675) and case–control study (Egger’s test: *p* = 0.752). Indicating no publication bias in this meta-analysis. Removing the included literatures one by one did not affect the results of this study, which means that the above results are stable and reliable.

## Discussion

This is the first meta-analysis evaluating the effects of C-section on the AR risk of offspring in child. Our systematic review revealed 22 studies reporting on cesarean delivery and offspring AR risk. Findings suggest that the overall pooled OR for AR in children was 1.19(*95% CI*: 1.12–1.27, *P* < 0.001).

Results from the meta-regression analysis revealed that sample size and publication year affected the association between C-section and AR, which explained nearly 38% and 19% of the variability between studies. We observed larger effects of studies with sample sizes smaller than 10,000. The current analysis finds evidence of a negative correlation between sample size and effect size in studies that get included in meta-analyses^56^. In a large sample size study, there may be people whose children have AR but have not reported or lost to follow up, resulting in an effect value that is lower than the actual^57,58^. In the subgroup analysis, although the effect value of the cohort study is lower than the whole studies, the overall statistical difference can also be explained in the follow-up time. The study found that the study with a follow-up time of less than 5 years has a larger effect value than the overall study. The small trials were of low quality of methodology compared with large trials which may partly account for the small-study effects and overestimating effect sizes. Meanwhile, meta-regression on publication year revealed that the publication year was also a factor contributing to the higher heterogeneity in this study. Higher effect sizes were found in literature published in earlier years (2000–2009). There are several possible explanations for this finding. Firstly, it is possible that earlier literature relied more on self-made questionnaires for the assessment of AR. This could have led to the inclusion of children who had not yet developed AR, thus affecting the consistency of the results. With the passage of time, the diagnosis and assessment methods for allergic rhinitis have continuously improved and been standardized. This was also explained in our subgroup analysis of the diagnostic mode for AR. Additionally, it is likely that the heterogeneity in the results is also influenced by the advancements in research methods and medical technologies in different time periods. These changes may include more accurate and standardized data collection methods, as well as the utilization of more advanced medical technologies and treatment methods. These factors could contribute to increased heterogeneity in the results across different time periods. In conclusion, after incorporating the adjusted effect size finding. We had gotten a conclusion that the overall risk of AR in children delivered after C-section increased by 19% and increased by 82% after C-section with parental history of allergy. In addition, elective C-section had a higher risk of AR compared with the whole study.

We had observed that the overall risk of AR in children delivered through C-section increased. This is consistent with the results of previous studies on C-section and allergic diseases. Previous studies have shown that C-section increase the risk of asthma, food allergies, and allergic dermatitis in children of offspring^59–61^. In our subgroup analysis with a family history of allergies, we found that C-section in families with a history of allergies increased the risk of children developing AR, with heterogeneity reduced to 0%. One possible explanation for the reduced heterogeneity within this subgroup is the shared genetic predisposition to allergies among family members. This shared genetic background could contribute to a more consistent pattern of allergic reactions and therefore a lower heterogeneity in the subgroup. In addition to genetic factors, families often share similar living conditions, dietary habits, and exposure to potential allergens environmental influences within families which could contribute to the reduced heterogeneity. A family history of allergy is a common risk factor for allergic diseases in offspring. C-sections with a family history of allergy have a higher risk of AR in children than overall C-sections. This demonstrates that the interaction between genes and the environment plays an important role in the development of such diseases^62^.

Many studies have shown that delivery by C-section is associated with an increased risk of disease associated with allergic disease in the offspring, but these studies have generally not discriminated between the effect of acute and elective C-section. In our study, we observed that children who were born by elective C-section had a higher chance of developing AR. Similar results were found in other studies. A cohort study conducted in the Denmark included over 750,569 participants and showed that elective C-section was higher than the effect of emergency C-section on the risk of asthma^63^. In Behzad Darabi’s meta-analysis. They also found that children delivered by elective C-section have a higher risk of asthma than children delivered by emergency C-section^64^.

The etiology behind C-section leading to AR in children is not fully understood until now. Based on the hygiene hypothesis, the rapid progression of the epidemic was attributed to a reduced diversity of early environmental microbe exposure, which decreased the occurrence of allergic diseases, including eczema, AR, and asthma^65^. In a cohort study of allergic diseases in South Africa^66^, the incidence of AR in urban children (25.3%, 95% *CI*: 22.8–27.8%, *P* < 0.001) was significantly higher than that in rural children (40.5%, 95% *CI*: 37.7–43.4%, *P* < 0.001). The rate of C-section in urban areas was twice as high as that in rural areas. This may be because the antenatal and childhood contact with farm animals in a rural environment resulted in low prevalence of AR in children owing to multiple exposures that mediate strong protective epigenetic modification through the microflora diversity^67–69^. Healthy or ‘tolerant’ individuals often produce high amounts of allergen-specific IgG4 and/or IgA, and IgA-deficient individuals are at risk of developing an allergy^70^. Generally, the exposure to microorganisms results in lipopolysaccharide stimulation of Toll-like receptors to produce interleukin 12 (IL-12) and interferon λ (IFN-λ), which promote the development of the Thnaive cells into Th1 effector cells. An impaired regulation of this immune pathway to children born by C-section^71^. Factors that alter the composition and diversity of the microbiome, such as the mode of infant delivery have a strong effect on immune responses, altering the immune response from one what is consistent with a state of tolerance to a dysfunctional hyper-responsive state that is associated with allergic diseases. However, conflicting results have been observed in recent epidemiological investigations. First, the incidence of AR began to decline in some Western European countries, but there was no obvious evidence that it was due to the decline in sanitary condition or increase in the number of family members^72^. Second, in a cohort study by Weber^73^, on personal and family cleanliness and allergic disease, which included 399 participants, dust parameters in the household objectively reflected personal cleanliness, including hand washing and household cleanliness, although personal cleaning or household cleanliness was not associated with the risk of allergy. Other microbial components in the indoor dust that were not affected by personal hygiene might play an important role. Considering the reasons for the increasing prevalence of AR in children based on the hygiene hypothesis, further studies are needed to determine the underlying genetic and epigenetic mechanisms, focusing on the role of early allergens and differences in epidemiological studies^74^.

At the same time, some studies have also shown that differences in the colonization of neonatal intestinal flora caused by caesarean section are also related to allergic diseases in offspring. The developmental starting point of the infant gut microbiota is still unknown, but undoubtedly, the process of delivery seems to be a key point in the development of the neonatal microbiota. Babies born via C-section miss out on this initial exposure to the mother's microbiota. Instead, they are colonized primarily by the skin and environmental microorganisms present in the operating room. The intestinal microflora of infants born through VD had a higher abundance of Bifidobacterium, Lactobacillus, Escherichia coli, and Bacteroides; demonstrating greater intestinal microbial diversity^75^. Conversely, the microbiome of infants born via C-section shows an increased prevalence of either skin flora or potentially pathogenic microbial communities such as Klebsiella, Enterococcus, and Clostridium^76^. There are several explanations for this mechanism. Firstly, Shohei Akagawa^77^ points out that children born by C-section have characteristics such as reduced Bacteroides and decreased butyric acid concentration in the gut. The decrease in butyric acid concentration inhibits the differentiation of immature T cells into Tregs, which are crucial for suppressing excessive immune responses. This impairment of the immune system's ability to suppress excessive immune responses can lead to the development of allergic diseases. Secondly, there was evidence that low α-diversity and relative abundance of particular gut-commensal bacteria genera are associated with childhood respiratory diseases. However, the higher relative abundance of Bacteroidaceae, Clostridiaceae, and Enterobacteriaceae, and the lower relative abundance of Bifidobacteriaceae and Lactobacillaceae were associated with the development of allergies^78^. In addition, our study has shown that elective C-section is associated with a higher risk of AR. This might be because the newborns in contact with C-section related contaminants were exposed to the maternal genital tract microbiome due to factors, such as premature rupture of membranes, resulting in partial restoration of the newborn’s bacterial community to levels similar to those of babies born through VD^79^. In conclusion, the mode of delivery is a significance factor affecting the composition of intestinal flora in newborns and infants, which plays an important role in the development of the immune system.

A large number of studies have consistently observed that C-section could lead to an increased incidence of AR in children, therefore, most of the experiments were observational studies on the effects of C-section on allergic diseases in children. Several societal factors that may contribute to the rise in C-section rates include fear of VD among pregnant women, doctors' desire to avoid unknown medical risks, and cultural and religious differences in certain regions. Therefore, in order to reduce the C-section rate, it is recommended to consider the individual circumstances and medical indications for each delivery, including but not limited to strictly defining the indications for cesarean section, promoting VD, widespread adoption of painless delivery techniques, and encouraging women who have had a cesarean section to attempt vaginal birth after cesarean. Factors other than C-section might also affect the development of allergic diseases in children, including race, birth season, geographical location, social class or income level, tobacco exposure during pregnancy, changes in an indoor environment, adverse pregnancy outcomes, dietary changes, and antibiotic use^15,18,80,81^, which would cause the failure of immune tolerance and increase the risk of AR. In response to their preventive measures, Lunjani^82^ suggested that microbial exposure was the most effective way to prevent atopic diseases within 1–2 years after birth. For example, the orderly combination of infant intestinal microbial ecosystem was formed by an exposure to the maternal microflora, breastfeeding, and early exposure to various food, and environmental microorganisms during the prenatal period, childbirth, and delivery^83^. However, in a cohort study of 335 children by Jelding-Dannemand^84^, exclusive breastfeeding was observed to be ineffective in reducing early childhood sensitization or high-risk childhood related diseases at the age of 7 years. We found that most of the literature has corrected for breastfeeding, whether breastfeeding reduce the risk of C-section-related AR in our study remains unelucidated. However, the early introduction of supplementary foods, especially cereals, fish, and eggs, appeared to prevent asthma and allergies, and the introduction of supplementary foods as early as possible while continuing breastfeeding might be a more important strategy to prevent allergies in children^85^. These included studies did not specifically investigate and adjust the diet of mothers during pregnancy and children. It is necessary to study these factors in future research to explore whether diet can reduce the risk of AR in children born by C-section.

This study has several limitations. First, in the subgroup analysis regarding different types of C-section, we identified higher heterogeneity. Most of the studies investigating the relationship between C-section and AR in children were retrospective and did not include specific clinical indications for mothers choosing elective or emergency C-sections. Preterm delivery and low birth weight^81^ may affect the development of children's immune system, but some studies are aimed at term pregnancy, normal birth weight newborns as inclusion criteria, and the specific reasons for choosing C-section have not been described in the article, such as fetal distress, placenta previa, umbilical cord prolapse. Second, moderate-high heterogeneity across studies was detected for C-section and AR. Different issues could generate this heterogeneity, including different study population, study location, sample size, duration of follow-up, outcome diagnosis, publication areas, number of years, and treatment of confounding factors. Subgroup analysis and meta-regression were run to deal with the heterogeneity by using random-effect models. Third, this study included few small-scale studies and self-reported AR for the diagnosis, self-report or information from parental interviews may be liable to recall bias, which might have caused the actual prevalence to be higher than the present results, thereby reducing the universality of our findings, any deviations or inaccuracies in individual studies could have also influenced our analysis.

Therefore, C-section increase the risk of AR in children compared with VD. In the case of a high C-section rate worldwide, formulating specific clinical guidelines and implementing appropriate management plans helping reduce the AR risk in children after C-section need to be strengthened.

## Conclusion

In conclusion, our study identifies and characterizes that C-section is associated with subsequent childhood AR. However, the confounding effects of delivery mode and Indications for C-section remain to be studied in more detail. Future well-designed studies are warranted to validate the findings of the current analysis. When the C-section rate remains high, it is suggested that the formulation of specific clinical guidelines and implementation of appropriate management plans can reduce the risk of AR.

## Data Availability

All data generated or analysed during this study are included in this published article.

## Abbreviations

AR: Allergic rhinitis
C-section: Cesarean section
OR: Odds ratio
95% CI: 95% Confidence interval
ISAAC: International Childhood Asthma and Allergy in Childhood
WHO: World Health Organization
VD: Vaginal delivery
NOS: Newcastle–Ottawa Scale
PRISMA: Preferred reporting items for systematic reviews and meta-analyses
